# Inhibition of aluminum chloride-induced amyloid Aβ peptide accumulation and brain neurodegeneration by *Bougainvillea spectabilis* flower decoction

**DOI:** 10.22038/IJBMS.2021.58246.12940

**Published:** 2021-10

**Authors:** Omar ME Abdel-Salam, Marwa El-Sayed El-Shamarka, Eman R Youness, Nermeen Shaffie

**Affiliations:** 1 Department of Toxicology and Narcotics, National Research Centre, Cairo, Egypt; 2 Department of Medical Biochemistry, National Research Centre, Cairo, Egypt; 3 Department of Pathology, National Research Centre, Cairo, Egypt

**Keywords:** Aluminum chloride, Amyloid Aβ, Bougainvillea spectabilis- flowers, Interleukin-6, Neurodegeneration, Oxidative stress, Paraoxonase

## Abstract

**Objective(s):**

To investigate the potential therapeutic effect of *Bougainvillea spectabilis* flower decoction on aluminum chloride (AlCl_3_)-induced neurotoxicity.

**Materials and Methods:**

Rats received daily intraperitoneal injections of AlCl_3_ at 10 mg/kg for two months and were treated with *B. spectabilis* decoction at 50 or 100 mg/kg or saline during the 2^nd^ month of the study. The control group received saline. Brain malondialdehyde (MDA), nitric oxide (NO), reduced glutathione (GSH), acetylcholinesterase (AChE), amyloid Aβ peptide, and interleukin-6 (IL-6) concentrations and paraoxonase-1 (PON-1) activity were determined and brain histology was done. Behavioral and neurological testing included Morris water maze (WMZ), Y maze, and wire hanging.

**Results:**

Compared with saline controls, AlCl_3_ significantly increased brain MDA and NO along with decreased GSH and PON-1 activity. It also increased AChE, IL-6, and amyloid Aβ concentrations. AlCl_3_ impaired motor strength and memory performance and caused brain neurodegeneration. *B. spectabilis* decoction given at 50 or 100 mg/kg protected against the biochemical and histopathological alterations evoked by AlCl_3_ by alleviating the increase in MDA and NO, and decrease in GSH and PON-1 activity. *B. spectabilis* decoction showed no significant effect on AChE but markedly decreased IL-6 and amyloid Aβ in the brain of AlCl_3_-treated rats. It also restored memory performance and motor strength, and protected against AlCl_3_-induced neurodegeneration.

**Conclusion:**

These results suggest that *B. spectabilis* flower decoction might prove of value in the treatment of Alzheimer’s disease.

## Introduction

Alzheimer’s disease is the most common neurodegenerative disorder in old age and the leading cause of dementia in late-life which leads to significant socioeconomic burden and reduced quality of life (1). The disease is primarily sporadic, but it also occurs in rare familial forms (2). The sporadic form accounts for about 95% of cases (3). Loss of recent memory and difficulty in acquisition of new information can be seen in the early stages of Alzheimer’s disease. This can be followed several years later by profound memory loss, cognitive decline, and behavioral changes, leading ultimately to death within 7-10 years of diagnosis (4, 5). The neuropathologic hallmarks of the disease are the presence of senile or β-amyloid plaques, neurofibrillary tangles along with neuronal and synaptic loss, and brain atrophy. The senile plaques are extracellular deposits of amyloid-beta (Aβ40 and Aβ42) peptides resulting from the abnormal processing of amyloid precursor protein by β- and γ-secretases. The neurofibrillary tangles are intracellular aggregates of the hyperphosphorylated microtubule-associated protein tau. In Alzheimer’s disease, there is also loss of cholinergic neurons of the basal forebrain. It is largely thought that the accumulation of Aβ peptides represents an important early event in the cascade of events leading to neuronal death in Alzheimer’s disease (6, 7). In this context, evidence suggests that the most toxic species are soluble Aβ oligomers that trigger oxidative stress and inflammation (8). The main neurochemical deficit in Alzheimer’s disease is reduced acetylcholine content and choline acetyltransferase activity in the nucleus basalis of Meynert and hippocampus (9). Therefore, cholinesterase inhibitors that increase the availability of acetylcholine and cholinergic activity are clinically used to treat patients with Alzheimer’s disease. There is evidence that the centrally acting cholinesterase inhibitors donepezil, rivastigmine, and galantamine are effective in improving cognition in mild to moderate disease (10). 

Presently, few drugs are available to ease the life of patients with Alzheimer’s disease and there is a small opportunity to find more than a few remedies by the year 2025 (1). It is clear, therefore, that there is a need for increased research efforts to find new therapeutics to treat Alzheimer’s disease.

There is increasingly available evidence from human and animal studies that implicates exposure to aluminum (Al) in the pathogenesis of sporadic Alzheimer’s disease (11, 12). The metal has widespread use in building and construction, electrical conductors and equipment, aluminum foil, cans, antacids, enteric-coated aspirin, and deodorants (13). A significant increase in Al was found in the brain from sporadic and familial forms of Alzheimer’s disease compared with controls that had no evidence of neurodegeneration (12). Moreover, Al was detected in amyloid fibers in cores of senile plaques in the hippocampus and temporal lobe of patients with Alzheimer’s disease (14). Animal studies have shown that exposure to Al salts caused oxidative stress, neuroinflammation, memory deterioration, and β-amyloid deposits similar to those found in Alzheimer’s disease (11, 15). Mice exposed to aluminum sulfate in drinking water developed Aβ deposits in their cerebral cortex (16) while IP injection of AlCl_3_ resulted in cortical atrophy, neuronal shrinkage, and amyloid deposition in the rat brain (17). Mice receiving subcutaneous injections of AlOH_3 _showed evidence of increased apoptosis of motor neurons and reactive astrocytes and microglia in the spinal cord and cerebral cortex (18). Studies also showed a significant increase in acetylcholinesterase (AChE) activity in the brain (19) and serum (20) of AlCl_3_-treated rodents. AChE is the main enzyme responsible for ACh breakdown, which results in termination of ACh action in the neuronal synapse (21). The Al-based rodent model for Alzheimer’s disease, therefore, is useful in the search for possible therapeutic interventions for Alzheimer’s disease (22).

*Bougainvillea* (family: *Nyctaginaceae*) is a popular ornamental plant used for decorating gardens because of its colorful flower bracts of red, pink, white, and yellow varieties. It is grown in many parts of the world in tropical and subtropical areas (23). The most widely available species are *Bougainvillea buttiana, Bougainvillea glabra, Bougainvillea spectabilis, *and *Bougainvillea peruviana* (24). *Bougainvillea *species have been used in folk medicine. Nevertheless, there are very few studies of extracts of their flowers in experimental animals. These studies showed anti-inflammatory (25), hypoglycaemic, hypolipidemic (26), and neuroprotective activities (27). Methanolic/water extracts of *B. spectabilis* were shown to protect against brain, liver, and kidney damage caused by rotenone in rats (27). In the present study, we aimed to investigate the effect of flower decoction from* B. spectabilis* in the model of Alzheimer’s disease caused by AlCl_3_ administration in rats. The effect of *B. spectabilis* flower decoction was examined at the biochemical, behavioral, and histological levels.

## Materials and Methods

***Animals***

The study was carried out using male Sprague–Dawley rats (160-180 g). Animals were group-housed under temperature and light-controlled conditions and allowed free access to standard laboratory rodent chow and water *ad libitum* during the study. Animal procedures followed the recommendations of the Ethics Committee of the National Research Centre and the National Institutes of Health Guide for Care and Use of Laboratory Animals (publication no. 85-23, revised 1985). 

***Chemicals and reagents***

Aluminum chloride (Sigma-Aldrich, St Louis, MO, USA) was dissolved in distilled water and injected intraperitoneally (IP) daily at a dose of 10 mg/kg. The acetylcholinesterase (AChE) kit was purchased from NOVA (Bioneovan Co., Ltd., DaXing Industry Zone, Beijing, China). Rat amyloid beta-peptide 1-41 ELISA Kit was purchased from SinoGeneClon Biotech Co., Ltd. The Interleukin-6 ELISA kit was purchased from Glory Science Co, Ltd, Del Rio, TX, USA. Other chemicals and reagents such as 2-thiobarbituric acid, 5, 5’-dithiobis (2-nitrobenzoic acid), nitrate reductase, Griess reagent, and phenylacetate were obtained from Sigma Chemical Co. (St. Louis, MO, U.S.A.).

***Plant material and extraction***

**Figure 1:**
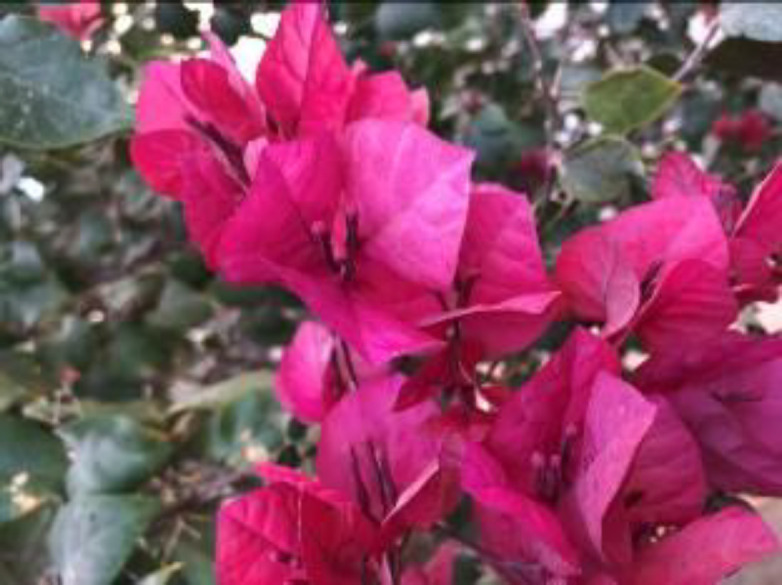
Flowers of *Bougainvillea Spectabilis *used in the study

The flowers of *B. spectabilis* (pink color) were obtained from a local garden in the Giza province (Figure 1). The plant was identified and authenticated by an expert in the Orman garden. The flowers of *B. spectabilis* (100 g) were cut into small pieces, crushed, and boiled in distilled water for 10 min. The decoction was then filtrated, concentrated by evaporation at 60 ^°^C using a rotary evaporator, and stored at 4 ^°^C. Rats were IP treated with *B. spectabilis* flower decoction at doses of 50 or 100 mg/kg. The doses of *B. spectabilis* were chosen based on previous studies that showed neuroprotective effects (27).

***Experimental design***

Rats were randomly assigned to different experimental groups, six rats each. Group 1 was treated with the vehicle (0.2 ml saline) daily for two months. Group 2 received daily IP injections of *B. spectabilis* flower decoction at a dose of 100 mg/kg. Group 3 received daily IP injections of AlCl_3_ at a dose of 10 mg/kg for two months. Groups 4 and 5 were administered AlCl_3_ (10 mg/kg, IP) daily for two months and were treated with *B. spectabilis* flower decoction at doses of 50 or 100 mg/kg (IP, daily) during the 2^nd^ month of the study. Morris water maze (WMZ), Y maze, and wire hanging tests were then carried out. By the end of the study, animals were killed by decapitation under light ether anesthesia, their brains were quickly removed out on an ice-cold plate, washed with ice-cold phosphate-buffered saline (7.4), weighed, and stored at -80 ^°^C for the biochemical studies. The tissues were homogenized with 0.1 M phosphate-buffered saline (pH 7.4) to give a final concentration of 10% weight/volume for the biochemical assays.

***Biochemical assays***

*Determination of lipid peroxidation*

Lipid peroxidation was determined in brain homogenates by measuring the level of malondialdehyde (MDA) according to Ruiz-Larrea *et al*. (28). In this assay, 2-thiobarbituric acid reacts with MDA at 25 ^°^C to yield a red-colored complex with a peak absorbance at 532 nm. 

*Determination of nitric oxide*

Nitric oxide was determined using the Griess reagent. Nitrate is converted to nitrite with the enzyme nitrate reductase. Nitrite then reacts with the Griess reagent to form a purple azo compound, and its absorbance is measured at 540 nm with a spectrophotometer (29). 

***Determination of reduced glutathione***

Reduced glutathione was determined in brain homogenates according to Ellman (30). Ellman´s reagent (DTNB; 5, 5’-dithiobis (2-nitrobenzoic acid)) reacts with the free thiol group of GSH to form 2-nitro-s-mercaptobenzoic acid. The chromophore has a yellow color and is determined with a spectrophotometer at 412 nm. 

***Determination of paraoxonase-1 activity***

The arylesterase activity of the paraoxonase-1 enzyme was determined by the use of phenylacetate as a substrate. In this assay, arylesterase hydrolyzes phenyl acetate forming phenol and the rate of hydrolysis was measured by monitoring the increase in the absorbance at 270 nm at 25 ^°^C with the use of a spectrophotometer. One unit of arylesterase activity is equivalent to 1 μM of phenol formed per minute. Enzyme activity expressed as kU/l is calculated based on the molar extinction coefficient of 1310 M-1 cm-1 for phenol at 270 nm, pH 8.0 and 25 ^°^C (31).

***Quantification of acetylcholinesterase***

Acetylcholinesterase (AChE) concentration was determined in supernatants using an ELISA kit purchased from NOVA (Bioneovan Co., Ltd., DaXing Industry Zone, Beijing, China) according to the manufacturer’s instructions.

***Quantification of amyloid Aβ peptide***

Rat amyloid beta-peptide 1-42 (Aβ 1-42) ELISA Kit (SinoGeneClon Biotech Co., Ltd) was used according to the manufacturer’s instructions.

***Quantification of interleukin-6***

The concentration of IL-6 in the brain was determined by a double-antibody sandwich enzyme-linked immunosorbent assay (ELISA) kit (Glory Science Co, Ltd, Del Rio, TX, USA) according to the manufacturer’s instructions.

***Behavioral testing***

*Water maze test*

In order to assess spatial working memory impairment after test compounds, the WMZ test was used (32). The apparatus consisted of a glass tank, narrowed to 20 cm wide, 40 cm in height, 70 cm in length, and filled to a depth of 21 cm with water maintained at 25 ^°^C. The escape platform was hidden from sight and placed 1 cm below the surface of the water. The time taken for each rat to escape onto the hidden platform was determined with a stopwatch and averaged for three consecutive trials. The cut-off time was 60 sec.

*Y-maze test*

This test estimates short-term memory. The apparatus is a metallic maze consisting of three arms: 40 cm long, 30 cm high, and 15 cm wide, extending from a central platform at 120^°^. At the beginning of the test day, each rat was placed in the middle of the maze and was left to explore its arms for 8 min. The sequence of entries into the arms was recorded. The rat with intact memory is expected to explore all of the 3 arms equally without repeating its entries. This is expressed in the form of a spontaneous alternations percentage, which was calculated using the formula [number of alternations/number of entries-2] X 100. The percentage of spontaneous alternation is considered a measure of spatial memory (33).

***Wire hanging test***

In order to evaluate motor strength, rats were hung by their forelimbs from a steel rod (25 cm long, 0.2 cm in diameter), 0.5 m above the bench. The time each rat could hang suspended from the rod was recorded for three trials with a cut-off time of 180 sec (34).

***Histopathological studies***

The brain tissues were fixed in a 10% neutral buffered formalin solution, embedded in paraffin, sectioned at a thickness of 5 μm, and then stained with hematoxylin and eosin (H&E). Sections were photographed and evaluated using a bright-field microscope (Optiphot 2; Nikon, Tokyo, Japan) for histological investigation.

***Statistical analysis***

Results are presented as mean±SEM. Data were analyzed with analysis of variance (ANOVA) followed by Duncan’s multiple range test for group comparisons. The Statistical Package for Social Sciences (SPSS) software (SPSS Inc, USA) was used.

## Results

***Biochemical results***

*Effect of Bougainvillea spectabilis decoction on lipid peroxidation*

**Figure 2:**
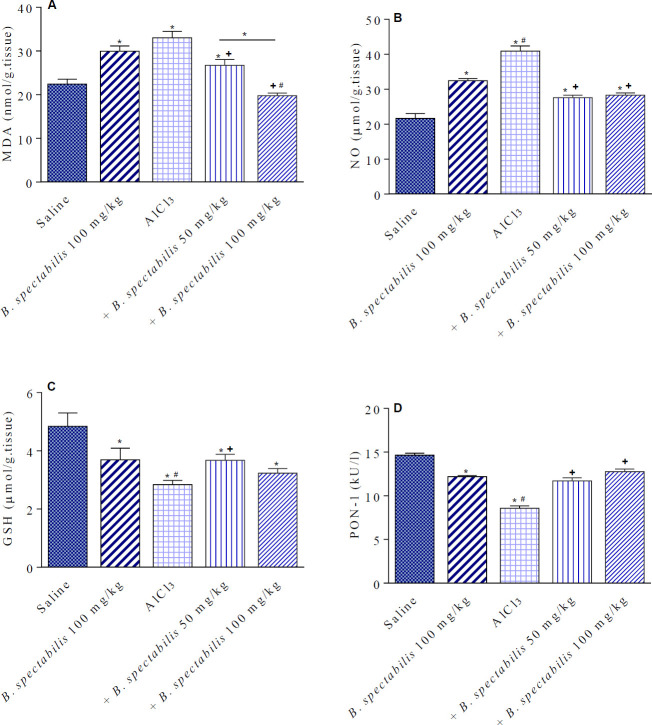
Effects of *Bougainvillea spectabilis* flower decoction on malondialdehyde (MDA), nitric oxide (NO), reduced glutathione (GSH), and paraoxonase-1 (PON-1) in the brain of rats treated with AlCl_3_. ^*^ *P<*0.05 vs saline and between different groups as shown in the figure. ^+ ^*P<*0.05 vs AlCl_3_ control. ^#^ *P<*0.05 vs flower decoction alone

Treatment with only* B. spectabilis *decoction at 100 mg/kg resulted in a significant increase in brain MDA by 33.4% relative to the saline control group (29.94±1.2 vs 22.45±1.1 nmol/g.tissue). AlCl_3 _caused significant increase in brain MDA by 47% (33.0±1.5 vs 22.45±1.1 nmol/g.tissue) compared with the saline control. *B. spectabilis *decoction given at doses of 50 or 100 mg/kg to AlCl_3_-treated rats significantly decreased brain MDA by 19.1% and 40.0% (26.7±1.38 and 19.8±0.59 vs 33.0±1.5 nmol/g.tissue) compared with the AlCl_3_ control group (Figure 2A). 

*Effect of B. spectabilis decoction on nitric oxide*

A significant increase in brain NO content by 49.7% (32.43±0.63 vs 21.66±1.41 µmol/g.tissue) was observed in rats given *B. spectabilis *decoction alone at 100 mg/kg. In rats treated with AlCl_3_, there was a significant increase in brain NO content by 88.9% (40.92±1.47 vs 21.66±1.41 µmol/g.tissue) compared with the saline control group. *B. spectabilis *decoction given at doses of 50 or 100 mg/kg to AlCl_3_-treated rats, significantly decreased brain NO by 32.5% and 30.8% (27.6±0.81 and 28.32±0.6 vs 40.92±1.47 µmol/g.tissue), respectively (Figure 2B).

*Effect of B. spectabilis decoction on reduced glutathione*

*B. spectabilis *decoction given alone at 100 mg/kg resulted in a significant decrease in brain GSH by 23.8% (3.69±0.4 vs 4.84±0.26 µmol/g.tissue). The level of GSH in the brain of AlCl_3_-treated rats showed a significant decrease by 41.3% (2.84±0.13 vs 4.84±0.26 µmol/g.tissue) compared with the saline control group. *B. spectabilis *decoction given at doses of 50 or 100 mg/kg to AlCl_3_- treated rats caused significant increase in GSH level by 29.2% and 13.7% (3.67±0.19 and 3.23±0.15 vs 4.84±0.26 µmol/g.tissue) compared with the AlCl_3_ control group (Figure 2C).

*Effect of B. spectabilis decoction on paraoxonase-1 activity*

There was a marked decrease in PON-1 activity by 16.8% in the group treated with *B. spectabilis *decoction alone at 100 mg/kg as compared with the saline control group (12.19±0.13 vs 14.66±0.22 kU/l). In AlCl_3_-treated rats, a significant decrease in PON-1 by 41.5% was observed as compared with the saline control group (8.58±0.26 vs 14.66±0.22 kU/l). *B. spectabilis *decoction, however, given at doses of 50 or 100 mg/kg to AlCl_3_-treated rats had significant increments in PON-1 activity by 36.4% and 48.7% (11.71±0.35 and 12.76±0.3 vs 8.58±0.26 kU/l) compared with the AlCl_3_ control group (Figure 2D).

*Effect of B. spectabilis decoction on acetylcholinesterase*

**Figure 3:**
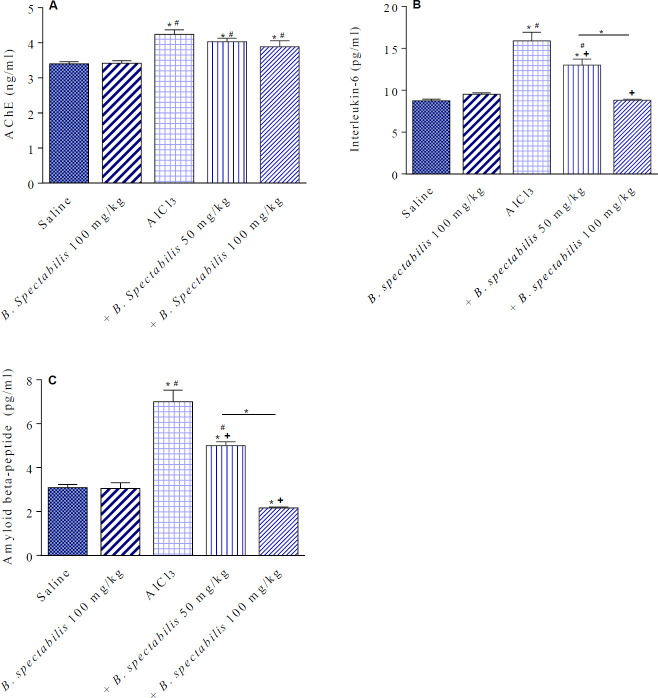
Effects of *Bougainvillea spectabilis* flower decoction on the level of acetylcholinesterase (AChE), interleukin-6, and amyloid Aβ peptide in the brain of rats treated with AlCl_3_. ^*^ *P<*0.05 vs saline and between different groups as shown in the figure. ^+^ *P<*0.05 vs AlCl_3 _control. ^#^ *P<*0.05 vs flower decoction alone

In saline-treated rats, *B. spectabilis *decoction given at 100 mg/kg had no significant effect on brain AChE concentration. Following repeated AlCl_3_ injections, AChE increased by 25.1% compared with the saline control value (4.24±0.13 vs 3.39±0.06 ng/ml). *B. spectabilis *decoction given at 50 or 100 mg/kg had no significant effect on brain AChE in AlCl_3_-treated rats (Figure 3A). 

*Effect of B. spectabilis decoction on interleukin-6*

*B. spectabilis *decoction given at 100 mg/kg to saline-treated rats had no significant effect on brain IL-6 concentration. IL-6 was significantly increased by 81.8% in AlCl_3_-treated rats (15.9±1.0 vs 8.74±0.21 pg/ml). IL-6 decreased by 18.2% and 44.6% in AlCl_3_-treated rats given B. spectabilis decoction at 50 or 100 mg/kg compared with the AlCl_3_ control group (Figure 3B).

*Effect of B. spectabilis decoction on amyloid Aβ peptide*

*B. spectabilis* decoction given at 100 mg/kg to saline-treated rats had no significant effect on brain amyloid Aβ concentration. Repeated administration of AlCl_3_ caused a significant increase in brain amyloid Aβ by 129.6% compared with the saline control group (7.0±0.53 vs 3.05±0.14 pg/ml). The level of amyloid Aβ in brains of AlCl_3_-treated rats was significantly decreased by 28.6% and 69.1% following treatment with *B. spectabilis *decoction at 50 and 100 mg/kg, respectively (Figure 3C).

***Behavioral*** ***testing***

*Effect of B. spectabilis decoction on memory performance*

*Water maze test*

**Figure 4:**
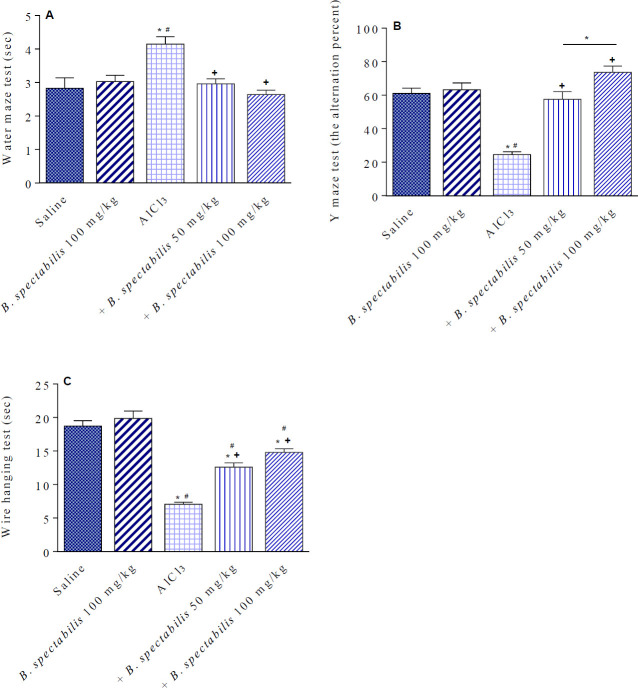
Effects of *Bougainvillea spectabilis* flower decoction on the AlCl3-induced impairments in the wire hanging, water maze, and Y maze tests. ^*^ *P<*0.05 vs saline and between different groups as shown in the figure. ^+^ *P<*0.05 vs AlCl3 control. ^#^ *P<*0.05 vs flower decoction alone

*B. spectabilis* decoction given alone at 100 mg/kg has no significant effect on the time spent by the mice to reach the platform. The escape latency, however, was significantly increased by 46.6% (4.15 vs 2.83 sec) in AlCl_3_-treated rats compared with the saline control group. Rats treated with AlCl_3_ /*B. spectabilis *decoction at 50 or 100 mg/kg showed escape latency times similar to those of the saline control group (Figure 4A).

*Y- maze test*

*B. spectabilis* decoction given at 100 mg/kg to saline-treated rats had no significant effect on the spontaneous alternation percentage. In AlCl_3_-treated rats, the alternation percentage decreased by 59.8% (24.5±1.7 vs 61.0±3.2 sec). The alternation percentage increased by 134.9% and 208.6% in rats treated with AlCl_3_/*B. spectabilis *decoction at 50 and 100 mg/kg compared with the AlCl_3 _control group (57.5±4.6 and 75.6±3.8 vs 24.5±1.7 sec) (Figure 4B).

***Effect of B. spectabilis decoction on motor strength in the wire hanging test***

*B. spectabilis* decoction given alone at 100 mg/kg did not impair the ability of rats in the wire hanging test. Compared with the saline control group, repeated injections of AlCl_3_ caused a significant decrease in the time the mouse spent in the wire-hanging test by 62.3% (7.05±0.31 vs 18.72±0.81 sec). Rats treated with AlCl_3_/*B. spectabilis *decoction at 50 or 100 mg/kg exhibited significant increases in the hanging time by 78.6% and 110%, respectively, compared with the AlCl_3 _control group (12.59±0.64 and 14.80±0.50 vs 7.05±0.31 sec) (Figure 4C).

***Histopathological results***

**Figure 5:**
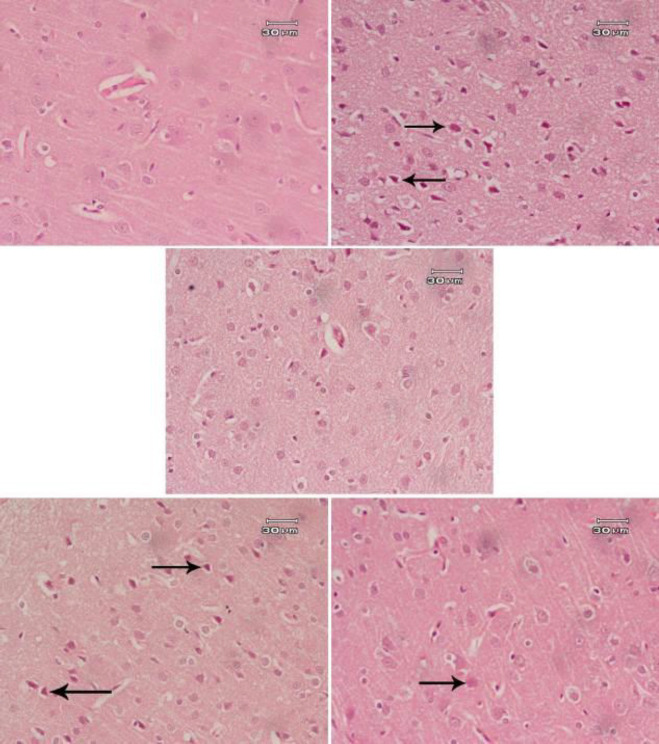
Photomicrographs of Hx & E stained cerebral cortex area from (A) Saline control showing normal structure. (B) AlCl_3_ only showing many small deeply stained neurons (arrow) denoting a degree of degeneration. (C) *Bougainvillea spectabilis* flower decoction (100 mg/kg) showing a close to normal structure. (D) AlCl_3_+*B. spectabilis* flower decoction (50 mg/kg) showing some small deeply stained neurons (arrow). (E) AlCl_3_+*B. spectabilis* flower decoction (100 mg/kg) showing that most neurons are normal in size and shape. Some cells are normal in size, but slightly deeper in color than normal (arrow)

**Figure 6:**
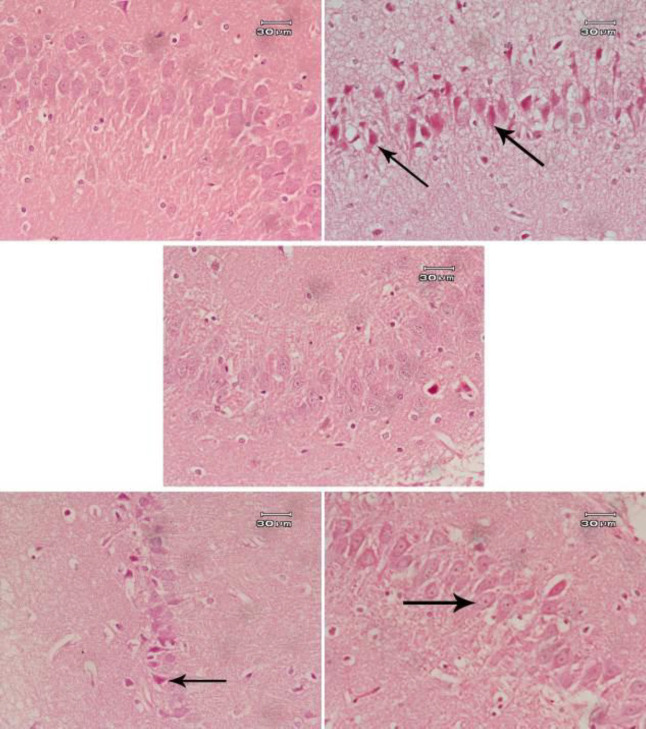
Photomicrographs of Hx & E stained hippocampus area from (A) Saline control showing normal neurons arranged in a band. (B) AlCl_3_ only showing most of the cells are small and deeply stained (arrow). (C) *Bougainvillea spectabilis* flower decoction (100 mg/kg) showing a close to normal structure. (D) AlCl_3_+*B. spectabilis* flower decoction (50 mg/kg) showing a decrease in thickness of the area, most of the neurons are still smaller in size, some of them are deeply stained (arrow). (E) AlCl_3_+*B. spectabilis* flower decoction (100 mg/kg) showing that most neurons are normalized showing large vesicular nuclei (arrow). An increase in thickness of this area is also observed

**Figure 7:**
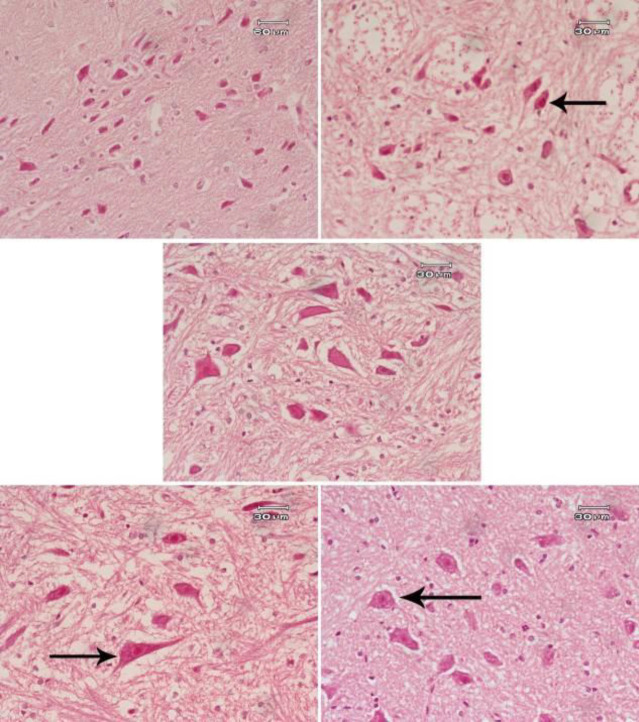
Photomicrographs of Hx & E stained sections of the substantia nigra from (A) Saline control showing the pigmented neurons scattered in this area of the brain. (B) AlCl_3_ only showing a remarkable decrease in size and number of pigmented neurons (arrow). (C) *Bougainvillea spectabilis* flower decoction (100 mg/kg) showing a close to normal structure. (D) AlCl_3_+*B. spectabilis* flower decoction (50 mg/kg) showing only a few pigmented neurons that regain their size (arrow). A decrease in the number of pigmented cells is still noticed. (E) AlCl_3_+*B. spectabilis* flower decoction (100 mg/kg) showing that many neurons are normal in size (arrow). An increase in the number of pigmented cells is also observed

Rats treated with saline showed normal structure of the cerebral cortex (Figure 5A), hippocampus (Figure 6A), and substantia nigra (Figure 7A). AlCl_3_ had neurotoxic effects; many neurons appeared smaller in size than normal and deeply stained (Figures 5B, 6B & 7B). Administration of *B. spectabilis* flower decoction alone at 100 mg/kg was very safe as the cerebral cortex, hippocampus, and substantia nigra were close to normal (Figures 5C, 6C & 7C). Rats subjected to AlCl_3_ and treated with the flower decoction demonstrated dose-dependent ameliorating effects on the damage induced by AlCl_3_ in these areas of the brain tissue (Figures 5D-E, 6D-5E, & 7D-E). 

## Discussion

This study aimed to investigate the therapeutic potential of *B. Spectabilis* flower decoction in an AlCl_3_-induced Alzheimer model in rats. Our results indicate that *B. spectabilis *decoction given to AlCl_3_-treated rats exerted anti-oxidant activity. *B. spectabilis *decoction markedly alleviated the increase in brain MDA and NO. It also increased GSH content and restored PON-1 activity. Additionally, *B. spectabilis *decoction decreased brain IL-6 or amyloid Aβ concentrations, normalized the behavioral/motor and memory deficits, and afforded neuroprotection. 

In this study, there was a significant increase in MDA levels in the brain of AlCl_3_-treated rats, which is suggestive of increased generation of ROS with a consequent attack on polyunsaturated fatty acids. Moreover, the GSH levels of the brain were also decreased following AlCl_3_. In brain tissue, glutathione (γ-L-glutamyl-L-cysteinylglycine) is the most abundant anti-oxidant that plays an important role in keeping the redox balance within the cell by shuttling between its reduced (GSH) and oxidized form (glutathione disulfide or GSSG). Glutathione also acts as a direct scavenger of superoxide, nitric oxide, hydroxyl radical, and peroxynitrite (35). The mechanism of Al-induced neurotoxicity involves oxidative stress (36). Studies showed that rodents treated with Al salts exhibited significantly increased brain lipid peroxidation and decreased anti-oxidants such as GSH, catalase, and superoxide dismutase activities (20, 37). Because Al has a fixed oxidation number, the metal cannot participate in redox reactions. Nevertheless, tissue oxidative stress induced by Al^3+^ is an indirect consequence of the displacement of iron from its binding sites. This would result in iron-mediated redox reactions, generation of ROS, and oxidative tissue damage (38). Studies showed that in liposomes and rat caudate/putamen homogenates, Al^3+^ stimulated lipid peroxidation initiated by melanin. This prooxidant effect of Al^3+^ might involve an interaction with superoxide (O_2_^-•^) derived from melanin autoxidation (39). The binding of Al^3+^ by O_2_^-• ^might also result in the formation of the oxidant aluminum superoxide semi-reduced radical ion (Al O_2_^2+•^) (36). Our results showed that *B. spectabilis* flower decoction was able to inhibit lipid peroxidation and increase GSH levels in the brain of AlCl_3_-treated rats. This suggests that the neuroprotective effect of *B. spectabilis* decoction against AlCl_3 _neurotoxicity involves an anti-oxidant activity. 

We demonstrated that the NO content in the brain of rats was markedly increased after Al^3+^, suggesting a possible role for nitric oxide in the neuronal damage induced by Al^3+^. Other studies have shown that AlCl_3_ caused a significant increase in NO level and increased expression of inducible NO synthase (iNOS) in the brain of rodents (17, 20). Moreover, it has been reported that specific inhibitors of either inducible or neuronal NO synthase were able to decrease oxidative stress and neuronal damage induced by AlCl_3_ (40). Nitric oxide produced in excessive amounts during inflammatory or toxic conditions causes neuronal degeneration. The mechanism is largely thought to be due to the formation of the strong oxidant peroxynitrite by the reaction of NO and O_2_^-• ^(41). Here we showed that the *B. spectabilis* flower decoction given to AlCl_3_-treated rats alleviated the increase in brain NO. This suggests that *B. spectabilis* decoction prevents AlCl_3_–induced neurotoxicity, at least in part, through an inhibitory action on NO generation.

Our results also showed marked decreases in PON-1 activity in the brains of AlCl_3_-treated rats, which is in agreement with previous studies (17, 20). Recent studies have shown that PON-1 activity declines in several neurodegenerative and neurological disorders. This suggested an important role for the enzyme in protecting neurons from oxidative stress (42). Because PON1 has important anti-oxidant and anti-inflammatory activities (43), the decline in its activity would render neuronal cells more susceptible to oxidative stress and inflammatory events, resulting in neurodegeneration. In the present study, treatment with *B. spectabilis* decoction was shown to restore PON-1 activity in the brain of AlCl_3_-treated rats. PON-1 activity is by inhibited oxidative stress (44). It is thus possible that the *B. spectabilis* decoction-induced increase in PON-1 activity is due to a lower level of oxidative stress.

One of the most important neuropathological findings in the brain of patients with Alzheimer’s disease is the accumulation of Aβ amyloid deposits in senile plaques that results from the abnormal processing of the amyloid precursor protein. These amyloid deposits initiate a cascade of oxidative and inflammatory events, ultimately leading to neuronal cell death (8, 9). In this study, the level of soluble Aβ peptides was significantly increased in the brain of AlCl_3_-treated rats compared with controls. Other studies have shown amyloid deposition in neurons in brain sections stained with Congo red from AlCl_3_-treated rats (17). In this work, we showed that *B. spectabilis* flower decoction was able to mitigate the increase in Aβ peptides. *B. spectabilis* decoction may inhibit the production and/or increase the degradation of Aβ peptides. In this context, it is worthy to mention that several studies indicated an anti-amyloidogenic activity for a number of flavonoids such as tannic acid, *curcumin*, and grape seed-derived polyphenols, preventing Aβ oligomerization and reducing Aβ production (45, 46). 

Our findings also showed increased AChE in the brains of AlCl_3_-treated rats. Al-induced cholinergic neurotoxicity is well established (47). Rats treated with AlCl_3_ (1 mg/kg, intravenously) exhibited increased AChE activity in caudate and decreased choline acetyltransferase in the basal forebrain and hippocampus (48). Other studies showed a marked increase of AChE activity in the brains and serums of rats given repeated IP injections of AlCl_3_ (10 mg/kg) for 6-8 weeks (17, 20). Rats treated with AlCl_3_ (10 mg/kg) for 12 weeks also showed increased AChE activity in their cerebral cortex and hippocampus (19). Moreover, an increase in AChE activity in different brain regions was observed after chronic administration of a low dose of AlCl_3_ in rats (49). In the present study, B. spectabilis decoction showed no significant effect on brain AChE in AlCl_3_-treated rats.

The present study also examined the effect of *B. spectabilis* flower decoction on motor/neurological alterations and memory following AlCl_3_ administration. Consistent with previous studies (20), AlCl_3_ resulted in impaired memory performance indicated by the increase in latency to escape to a hidden platform in the WMZ test and a decrease in the spontaneous alternation percentage in the Y-maze test. The impairment of spatial and working memory could be due to a decrease in acetylcholine levels in the cortex. Rats treated with AlCl_3_ also showed significant decreases in their grip power as evidenced by a shorter time to hang suspended from a steel rod as compared with saline controls. We found that *B. spectabilis* decoction normalized the memory deficits and grip weakness induced by AlCl_3_. It is suggested that the observed improvements in memory performance and motor strength by *B. spectabilis* decoction are the results of decreased brain inflammation (Il-6), oxidative stress, and Aβ peptide load. 

In our study, we investigated the therapeutic effect of* B. spectabilis* flower decoction on brain oxidative stress, inflammation, neurodegeneration, amyloid Aβ accumulation, and on behavioral/motor and memory deficits induced by administering AlCl_3_ to rats for two months. *B. spectabilis *decoction given at 100 mg/kg to saline-treated rats by itself induced an increased brain lipid peroxidation, NO along with decreased GSH level, indicative of increased oxidative stress, but with no histologic evidence of brain injury. *B. spectabilis* decoction alone had no effect on brain AChE, IL-6, amyloid Aβ concentration, motor strength, and impaired memory performance in saline-treated rats. However, B. spectabilis decoction given to rats that had been treated with AlCl_3_ for two months exerted anti-oxidant activity, markedly alleviating the increase in brain MDA and NO and depletion of GSH. *B. spectabilis* also markedly decreased IL-6 and brain amyloid Aβ concentrations. The effect of *B. spectabilis* on amyloid Aβ in the brains of AlCl_3_-treated rats is an intriguing observation and could be the result of decreased oxidative stress and neuroinflammation. Moreover, the decoction normalized behavioral/motor and memory deficits and afforded neuroprotection against AlCl_3_-induced neuronal damage. Our results, however, showed that* B. spectabilis* decoction had no significant effect on AChE in the brain of AlCl_3_-treated rats. *B. spectabilis *decoction alone thus showed pro-oxidant properties**,** but in the presence of oxidative stress, it exerted anti-oxidant effects. These results are in a major part in agreement with previous studies using methanolic/water extract of the *B. spectabilis *flower. In these studies, *B. spectabilis* flower extracts were shown to decrease brain lipid peroxidation, exert an antiapoptotic effect, decreasing the number of positively stained neurons with anti-caspase-3 antibody, and protecting against neuronal damage evoked by rotenone in rat brains. In absence of rotenone, the extracts, however, showed evidence of increased brain oxidative stress (27). *Bougainvillea *flowers contain several phenolic compounds such as gallic, ferulic, protocatechuic, chlorogenic, and caffeic acids and flavonoids including quercetin, myricetin, kaempferol, apigenin, and rutin (24). Studies indicated that these phenolics and flavonoids are important health maintaining compounds by virtue of their anti-oxidant activities that include direct scavenging of ROS, chelation of transition metal ions Fe^+^, Cu^+^**,** and regeneration of cell membrane anti-oxidants, e.g., vitamin E (50). These compounds, however, can be pro-oxidants at high concentrations or under certain circumstances (50, 51). This effect involves the generation of polyphenolic phenoxy radicals (52, 53). It has been suggested that dietary pro-oxidants, such as phytochemicals and fatty acids, increase ROS just to activate the transcription factor, nuclear factor, erythroid-2 related factor 2 (Nrf2), and heat shock factor, thereby, increasing the levels of anti-oxidant enzymes and heat shock proteins which protect against ROS. Thus, flavonoids with the potential to generate oxidative stress induce electrophilic-responsive elements and via activation of Nrf2 induce the expression of anti-oxidant enzymes that protect against oxidative stress (54).

## Conclusion

In summary, the present study indicated for the first time that *B. spectabilis* flower decoction was able to reduce brain oxidative stress, IL-6, and the accumulation of amyloid Aβ in the AlCl_3_-induced model of Alzheimer’s disease in rats. Moreover, *B. spectabilis* flower decoction restored memory and motor impairment and afforded histological neuroprotection. Our findings suggest that *B. spectabilis* flower decoction could be a possible therapy in the prevention and/or treatment of Alzheimer’s disease.

## Conflicts of Interest

The authors declare no conflicts of interest.

## Authors’ Contributions

OMEAS: Study conception and design and data analysis; MESES: preparation of plant material, performed experiments and data analysis; ERY: biochemical analysis; NS: histopathological studies and interpretation; OMEAS, MESES, ERY, NS: manuscript preparation, revision and final approval of the version to be published.
